# Isoflurane-induced neuroinflammation and NKCC1/KCC2 dysregulation result in long-term cognitive disorder in neonatal mice

**DOI:** 10.1186/s12871-024-02587-6

**Published:** 2024-06-05

**Authors:** Dongni Xu, Jiayi Liu, Shiyu Meng, Meixian Sun, Yuqing Chen, Yu Hong

**Affiliations:** 1 Department of Anesthesiology, Sun Yat-Sen Memorial Hospital, Sun Yat-Sen University, No. 107 Yanjiang West Road, Guangzhou, 510120 China; 2 The Eighth People’s Hospital of Qingdao, Qingdao, Shandong Province China

**Keywords:** Isoflurane, Cation-chloride cotransporters, Neonatal rats, Neuroinflammation, Bumetanide, Cognitive impairment

## Abstract

**Background:**

The inhalational anesthetic isoflurane is commonly utilized in clinical practice, particularly in the field of pediatric anesthesia. Research has demonstrated its capacity to induce neuroinflammation and long-term behavioral disorders; however, the underlying mechanism remains unclear [1]. The cation-chloride cotransporters Na^+^–K^+^–2Cl^−^–1 (NKCC1) and K^+^–2Cl^−^–2 (KCC2) play a pivotal role in regulating neuronal responses to gamma-aminobutyric acid (GABA) [2]. Imbalances in NKCC1/KCC2 can disrupt GABA neurotransmission, potentially leading to neural circuit hyperexcitability and reduced inhibition following neonatal exposure to anesthesia [3]. Therefore, this study postulates that anesthetics have the potential to dysregulate NKCC1 and/or KCC2 during brain development.

**Methods:**

We administered 1.5% isoflurane anesthesia to neonatal rats for a duration of 4 h at postnatal day 7 (PND7). Anxiety levels were assessed using the open field test at PND28, while cognitive function was evaluated using the Morris water maze test between PND31 and PND34. Protein levels of NKCC1, KCC2, BDNF, and phosphorylated ERK (P-ERK) in the hippocampus were measured through Western blotting analysis. Pro-inflammatory cytokines IL-1β, IL-6, and TNF-α were quantified using ELISA.

**Results:**

We observed a decrease in locomotion trajectories within the central region and a significantly shorter total distance in the ISO group compared to CON pups, indicating that isoflurane induces anxiety-like behavior. In the Morris water maze (MWM) test, rats exposed to isoflurane exhibited prolonged escape latency onto the platform. Additionally, isoflurane administration resulted in reduced time spent crossing in the MWM experiment at PND34, suggesting long-term impairment of memory function. Furthermore, we found that isoflurane triggered activation of pro-inflammatory cytokines IL-1β, IL-6, and TNF-α; downregulated KCC2/BDNF/P-ERK expression; and increased the NKCC1/KCC2 ratio in the hippocampus of PND7 rats. Bumetadine (NKCC1 specific inhibitors) reversed cognitive damage and effective disorder induced by isoflurane in neonatal rats by inhibiting TNF-α activation, normalizing IL-6 and IL-1β levels, restoring KCC2 expression levels as well as BDNF and ERK signaling pathways. Based on these findings, it can be speculated that BDNF, P-ERK, IL-1β, IL-6 and TNF - α may act downstream of the NKCC1/KCC2 pathway.

**Conclusions:**

Our findings provide evidence that isoflurane administration in neonatal rats leads to persistent cognitive deficits through dysregulation of the Cation-Chloride Cotransporters NKCC1 and KCC2, BDNF, p-ERK proteins, as well as neuroinflammatory processes.

**Supplementary Information:**

The online version contains supplementary material available at 10.1186/s12871-024-02587-6.

## Introduction

Worldwide, millions of infants (0–3 years of age) require anesthesia for surgery each year, and more than a quarter of them are exposed to general anesthesia within the first year of life. General anesthetic exposure can cause long-term cognitive impairment, especially in the developing brain, but its mechanism is still unclear [3].

Isoflurane is a volatile anesthetic that is usually maintained as a general anesthetic in various surgeries for both patients and animals. As reported, its anesthetic action can be affected by different mechanisms, such as agonist action on gamma-aminobutyric acid (GABA) receptors and antagonist action on N-methyl-D-aspartate (NMDA) receptors [4]. According to an increasing number of findings, neonatal animals may experience neuropathological changes after anesthesia and suffer abnormal social behaviors and cognitive dysfunction in the long term, but the mechanism is still unclear. The early use of such agent has the potential to evoke neurotoxic effects on future, hence, its mechanism should be deeply understood. NKCC1 excites GABA in immature neurons, but KCC2 expression inhibits GABA maturation.At the same time, studies have reported that persistent neuroinflammation caused by isoflurane is an important cause of cognitive impairment. Among the many inflammatory factors, IL-1βis considered to be the dominant factor in the persistence of neuroinflammation and is associated with cognitive impairment caused by isoflurane. However, the molecular mechanism by which IL-1βcauses cognitive impairment, especially in the early stages of development, is unclear [5].We aimed to investigate the mechanism of inflammation and chloride channel protein in isoflurane induced long-term cognitive dysfunction in neonatal mice.

## Materials and methods

### Animals

The animal experiments conducted in this study were approved by the Institutional Animal Care and Use Committee (Approval No: SYSU-IACUC-2022-000097) and the Laboratory Animal Ethics Committee of Sun Yat-sen University. Neonatal Sprague-Dawley rats were housed in polypropylene cages with regulated temperature, humidity, and a 12-hour light/dark cycle. All rats were provided ad libitum access to water and food.

### Anesthesia

We randomly assigned seventy postnatal Day 7 (PND7) SD mice, weighing between 13 and 18 g and of both sexes, to two groups: the anesthesia group (*n* = 35) received isoflurane for 4 h (2.5% for 3 min during induction; followed by 1.5% for 4 h in a carrier gas mixture of 21% oxygen and 79% nitrogen), while the control group (*n* = 35) received only air. The pups were kept warm and maintained at a constant normal temperature throughout the experiment. An infrared analyzer from Datex Ohmeda, USA was used to continuously monitor the chamber’s gas composition including isoflurane, oxygen, carbon dioxide concentrations, as well as the lowest alveolar concentration. Immediately after anesthesia, five rats from each group underwent arterial blood sample extraction through puncture of the left ventricle chest wall using an i-STAT blood gas analyzer from Abbott, USA for analysis of blood gases and a Bayer blood glucose meter (04680464002 model) from Bayer, USA for measuring blood glucose concentration. After confirming that NKCC1 may be associated with cognitive impairment in neonatal rats following isoflurane anesthesia, further research was conducted on an additional group receiving isoflurane anesthesia combined with bumetanide intraperitoneal injection (at a dose of 1.82 mg/kg), administered fifteen minutes prior to inhalation of isoflurane. To prevent dehydration after anesthesia completion, all anesthetized rats were subcutaneously injected with 500µL normal saline solution three hours later.

### Tissue isolation

**Fig. 1:**
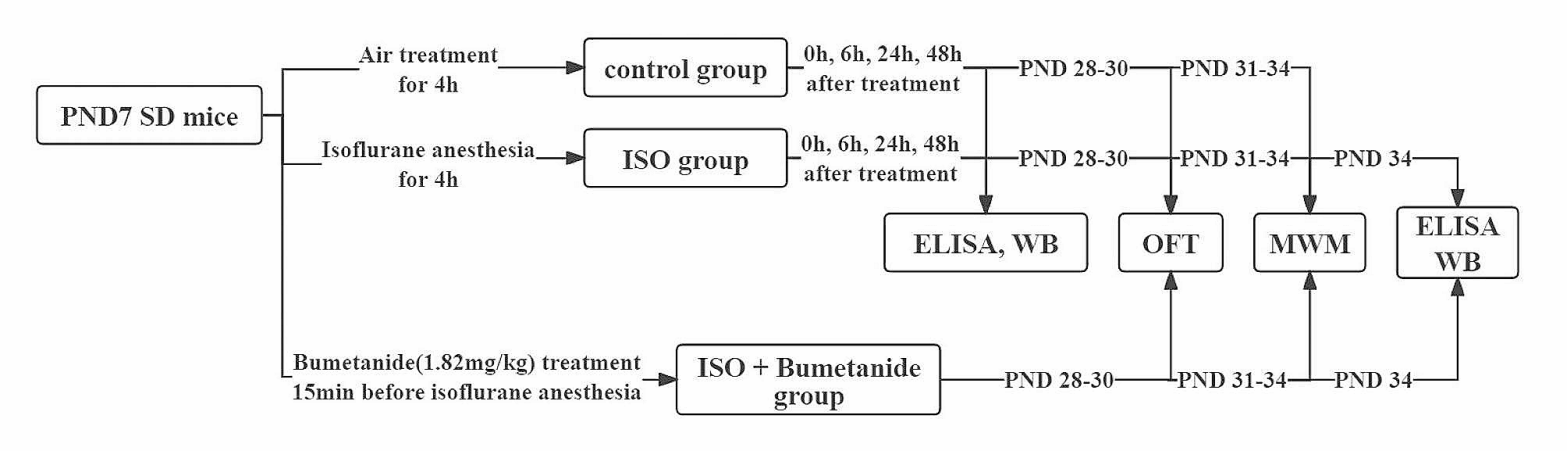
A concise schematic depiction of experimental procedures

We divided P7 rats into two groups: one group was exposed to isoflurane and the other group was exposed to air for a duration of 4 h. At 0, 6, 24, and 48 h after anesthesia termination, we sacrificed some of the rats and conducted Western analysis on NKCC1, NKCC2, BDNF, and p-ERK using half of their hippocampus. The other half was used for ELISA to measure the expression levels of pro-inflammatory cytokines TNF-α, IL-1β, and IL-6. Rat hippocampi were collected at four time points with five pups per time point (T1). Acute dysregulation of NKCC1 and KCC2 induced by sevoflurane exposure was immediately assessed at T2 (6 h), T3 (24 h), and T4 (48 h) after exposure to evaluate long-term protein changes. At the end of the experiment, we deeply anesthetized the pups with isoflurane followed by transcranial perfusion using ice-cold phosphate-buffered saline via the left ventricle. Brains were extracted and hippocampi were rapidly dissected on ice before being frozen in liquid nitrogen. Samples were stored at -80 °C while crude plasma membrane fractionation followed a standard protocol. The remaining ten mice in each group were returned to their home cages once they regained righting reflex. The remaining pups were allowed to grow freely until behavioral testing was performed 28 days after birth.The experimental timeline is illustrated in Fig. 1.

### ELISA (enzyme-linked immunosorbent assay) assay

We obtained 30 mg of hippocampal tissues from rats in each group and prepared them as 10% hippocampal homogenate using normal saline. Subsequently, the homogenates were centrifuged at 3000 rpm for 15 min. The resulting supernatant was collected, and the levels of TNF-α, IL-1β, and IL-6 in rat hippocampal tissues were determined using ELISA kits (Guangzhou, Weijia company).

### Western blotting

Frozen hippocampal tissues were extracted, homogenized, and lysed. The BCA protein quantitative method was utilized to determine the protein concentration. A 5× loading buffer was employed to mix the protein, which underwent denaturation treatment at 37 °C for 30 min. A separation gel of 10% and a concentrated SDS gel of 5% were used. PAGE electrophoresis was conducted at a constant current of 300 mA for 2.5 h. Rabbit anti-rat BDNF antibody (ABEAM, UK), rabbit anti-rat p-ERK antibody (CST, US), and mouse anti-rat β-actin antibody (Santa Cruz, US) were diluted at a ratio of 1:2000 and incubated overnight at 4 °C. Sheep anti-rabbit secondary antibody (Wuhan Bodeo Biological J: Cheng Co., Ltd.) or sheep anti-mouse secondary antibody (Wuhan Bodeo Biological Engineering Co., Ltd.) were added at a dilution of 1:5000 and incubated at room temperature. The film was developed using an ECL chemiluminescence detection kit. Subsequently, the film was scanned by a BIO-RAD gel imaging system and quantitatively analyzed using Quantity One software to determine the optical density values. The expression levels of KCC2, NKCC1, BDNF, and p-ERK were determined by calculating their respective ratios with β-actin based on optical density values.

### Open field test (OFT)

The open-field test was utilized to assess both general locomotor activity and anxiety-like behaviors [6]. Rats (*n* = 10) were gently placed in a rayless open-field apparatus measuring 50 cm × 50 cm × 37 cm, constructed with Plexiglas walls and a black floor, and allowed to freely explore for five minutes. Between each trial, the arena was cleaned using 70% ethanol. Data recording was facilitated by video footage, while EthoVision XT 7.0 (Noldus, Wageningen, Netherlands) aided in data analysis. Locomotor activity was measured based on distance traveled and speed, while evaluation of anxiety-like behaviors relied on frequency and duration of visits to the center zone.

### Morris water maze (MWM)

The Morris water maze test (MWM) was conducted on postnatal day 31 to assess the cognitive functions of rats in each group. The place navigation test was carried out for the initial three days, followed by a spatial probe test on the fourth day. Rats were released into the water facing the wall from a designated starting point in the pool, and their escape latency (i.e., time taken to find the fixed platform) was recorded. Rats were required to remain on the platform for 30 s; if they failed to locate it within 90 s, they were guided towards it and allowed to stay for 30 s. On the fourth day, we removed the platform from the water and observed both crossing times over its previous location and duration of staying in that quadrant.

### Statistical method

SPSS 21.0 software was utilized for conducting the statistical analysis. Data measurement was performed using the mean ± standard deviation (x ± s). Group comparison was carried out using Student’s t-test. Repeated measurements t-test were employed to analyze significant differences between different groups, and post hoc testing was conducted using Tukey’s HSD test. A significance level of *P* < 0.05 indicated statistically significant differences.

## Results

### Isoflurane anesthesia leads to long cognitive impairment in neonatal rats

**Fig. 2:**
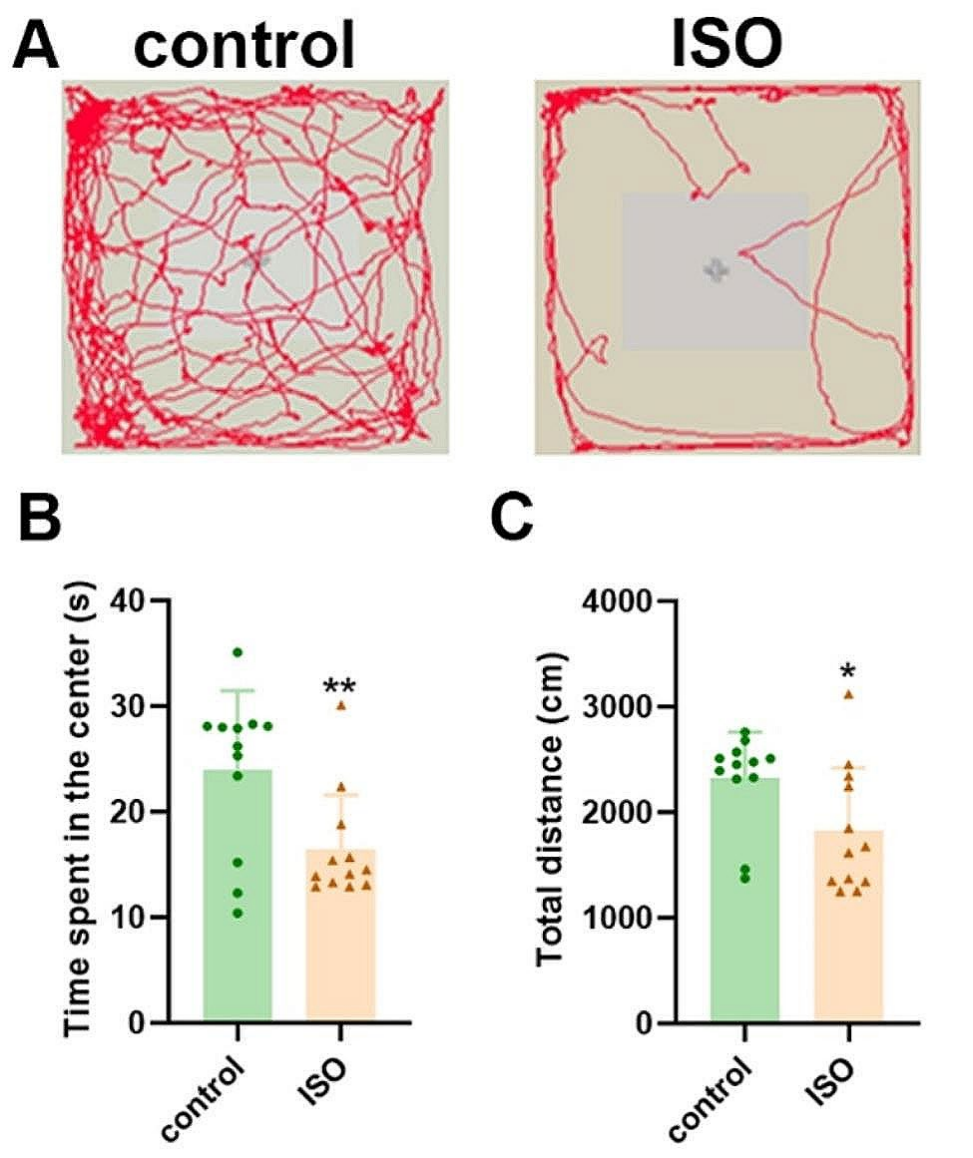
**A** Movement trajectories of mice in the CON and ISO groups were analyzed in the open field test experiment. **B** The time spent in the center of the open field test was compared between the two groups. **C** Total distance covered during the open field test was measured for both groups. The data are presented as mean ± standard deviation (*n* = 12 mice per group). Statistical significance was determined using *p < 0.05 compared to the CON group, and **p < 0.01 compared to the CON group

The open-field test was conducted to assess the locomotor activity and emotional behavior of rats. It is evident that the ISO group exhibited reduced locomotion trajectories in the central region, with a significantly shorter total distance compared to CON pups. Relative to the control group, isoflurane-treated rats spent less time exploring the center area. We consider these reductions in exploration time and entry frequency as indicators of anxiogenic-like behavior, suggesting that offspring treated with isoflurane also displayed anxiety-like behavior. The detailed data are presented in Fig. 2.

**Fig. 3:**
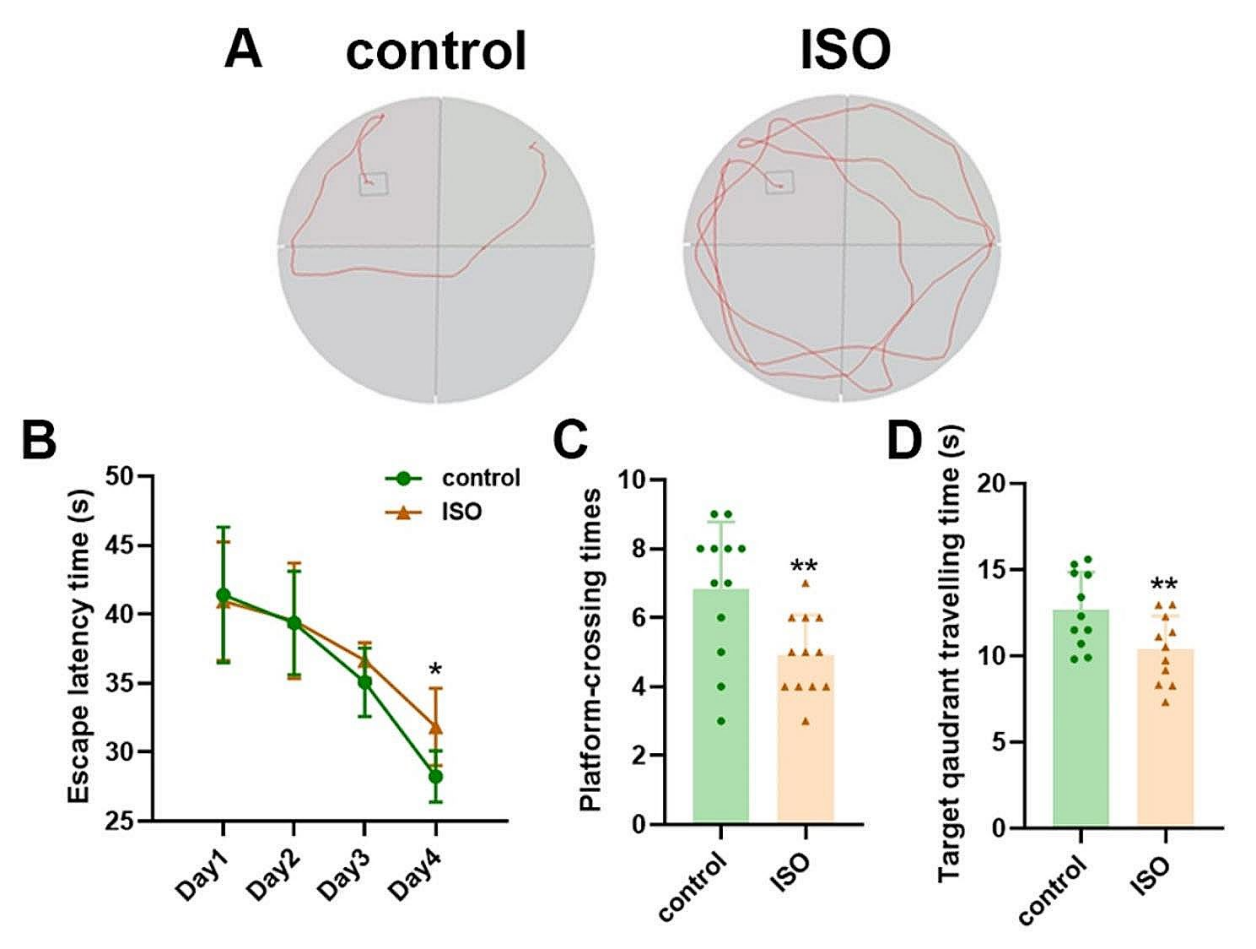
**A** The trajectory diagram of the CON and ISO groups in the platform finding experiment clearly indicated that the ISO mice required more time to locate the correct platform position. **B** The escape latency time of both mouse groups in the MWM navigation test revealed that on the fourth day, ISO mice took longer to find the platform. **C** The number of times each group crossed over the platform during the probe trial of the MWM test was analyzed. **D** In comparison to the CON group, it was evident that target quadrant traveling times were significantly shorter for mice in the ISO group. Data are presented as means ± SEMs (*n* = 12 per group) and were statistically compared using repeated measure 2-way ANOVA. ∗*P* < 0.05, control vs. ISO

The Morris water maze (MWM) was utilized to evaluate rodents’ learning and spatial memory abilities by providing distal cues for navigating from various start points surrounding an open swimming arena towards a submerged escape platform. Extensive evidence supports its validity in measuring hippocampal-dependent spatial learning and memory [7]. Control pups performed well in the MWM task; however, rats treated with isoflurane took longer to reach the platform, indicating impaired performance. Additionally, isoflurane administration resulted in a decreased percentage of crossing time during the MWM experiment compared to control rats at PND32, suggesting long-term detrimental effects on memory function caused by isoflurane exposure. The detailed data are illustrated in Fig. 3.

### The inflammatory response of hippocampus contributes to cognitive impairment after neonatal isoflurane anesthesia

**Fig. 4:**
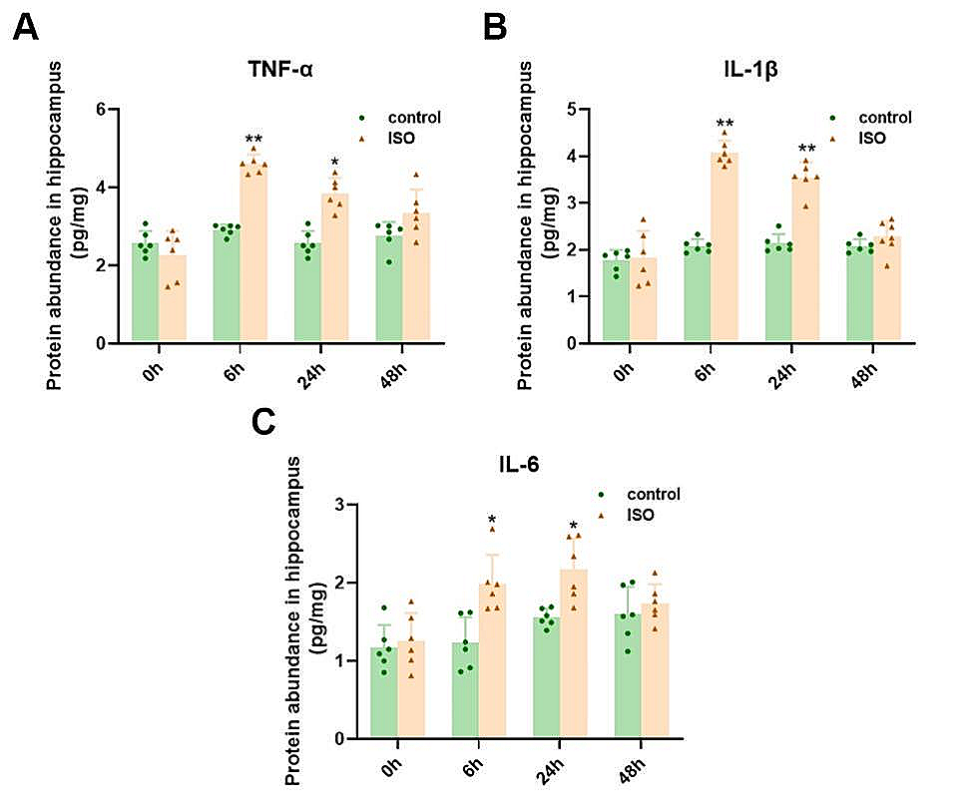
**A, B, C** Compared to the CON group, the concentrations of hippocampal inflammatory factors TNF-α, IL-1βand IL-6 were significantly elevated at 6 and 24 h. The data are presented as means ± SEMs (*n* = 6 per group) and were analyzed using a t-test. ∗*P* < 0.05,∗∗*P* < 0.01 control vs. ISO

Studies have demonstrated that persistent neuroinflammation induced by isoflurane serves as a significant contributor to cognitive impairment. In order to investigate alterations in inflammatory factors following isoflurane treatment, ELISA was employed to assess the expression levels of proinflammatory cytokines IL-1β, TNF-α, and IL-6. Compared to the control group, the hippocampus of neonatal rats in the isoflurane group exhibited a notable elevation in pro-inflammatory cytokines IL-1β, TNF-α, and IL-6. The detailed data are illustrated in Fig. 4.

### Isoflurane anesthesia affects the expression of NKCC1/KCC2

**Fig. 5:**
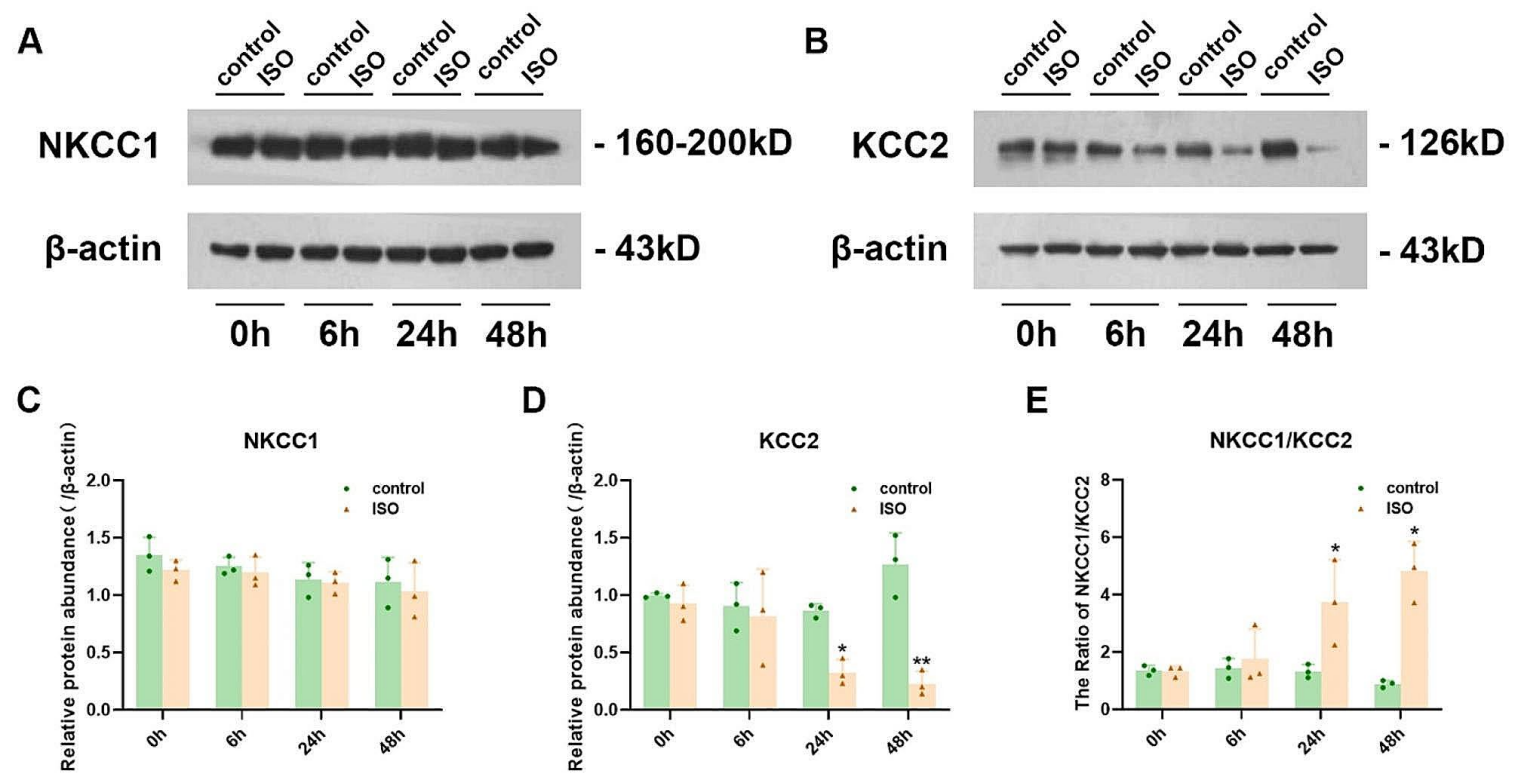
**A, B** The protein levels of NKCC1 and KCC2 in hippocampal tissue lysates at different time points (0, 6, 24, and 48 h) were determined using immunoblotting. **C, D, E** Quantification of the data shown in Figure A was performed. The data are presented as the mean ± SEM (*n* = 3 per group), with statistical significance indicated as *P < 0.05 and **P < 0.01 for control vs. ISO

**Fig. 6:**
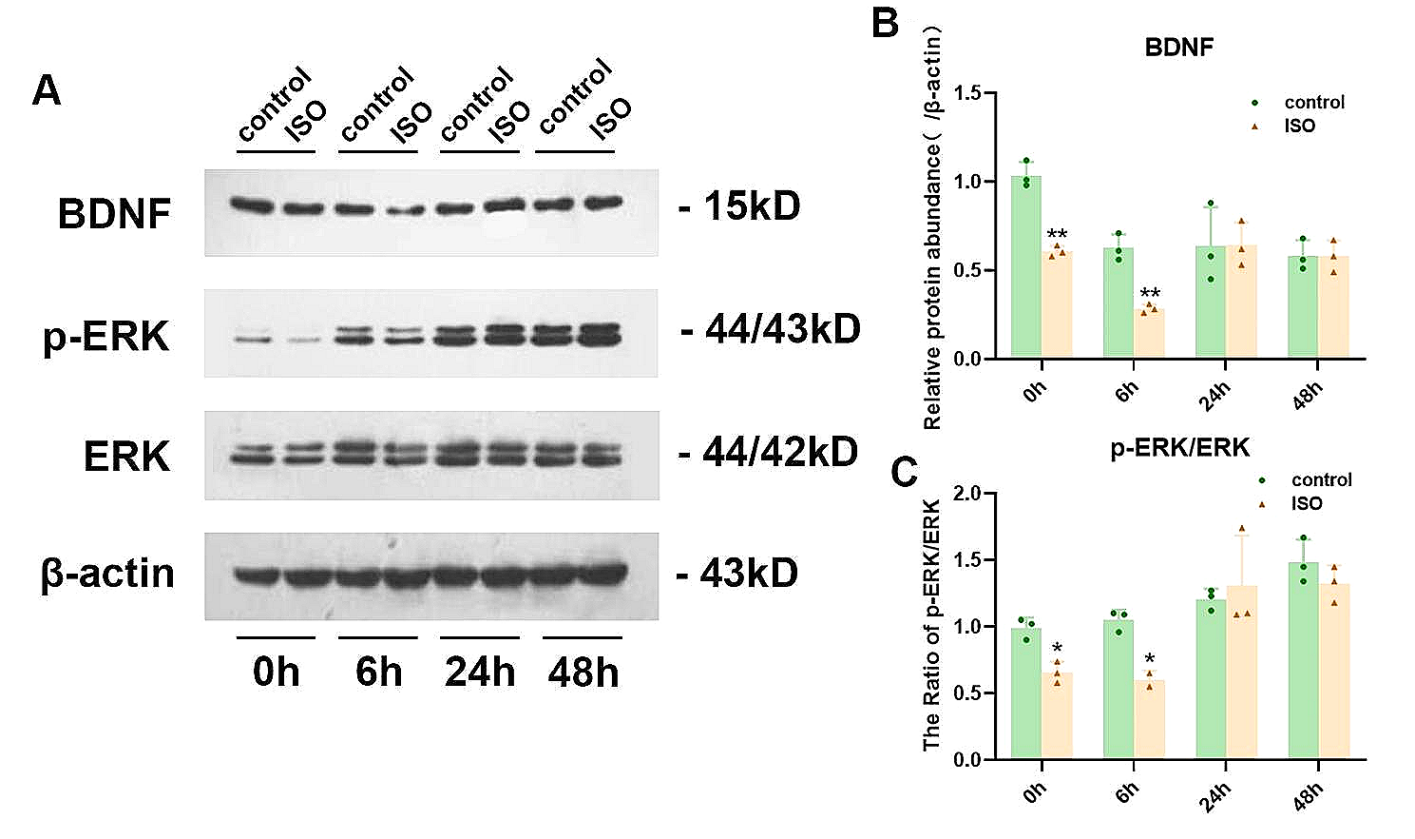
**A** Immunoblotting was employed to determine the protein levels of BDNF, P-ERK, and ERK in hippocampal tissue lysates at various time points (0, 6, 24, and 48 h). **B. C** Quantification of the data depicted in A was performed. The data are presented as mean ± SEM (*n* = 3 per group), with statistical significance denoted as ∗*P* < 0.05 and ∗∗*P* < 0.01 for comparisons between control and ISO groups

NKCC1/KCC2 imbalance alters GABA neurotransmission, potentially resulting in hyperexcitability of neural circuits and reduced inhibition following neonatal exposure to anesthesia. The protein expression investigation was conducted on the rat hippocampus at PND7 using western blotting at 0 h, 6 h, 24 h, and 48 h after anesthesia termination. KCC2 expression showed a decrease at 24 h and 48 h post isoflurane exposure, while the NKCC1/KCC2 ratio increased. BDNF and p-ERK expression in the hippocampus were downregulated at 0 h and 6 h, whereas there were no significant differences observed in NKCC1 expression across all time points examined. Figures 5 and 6 present all the data.

### NKCC1 specific inhibitor (bumetanide) alleviates cognitive impairment caused by isoflurane in neonatal rats

**Fig. 7:**
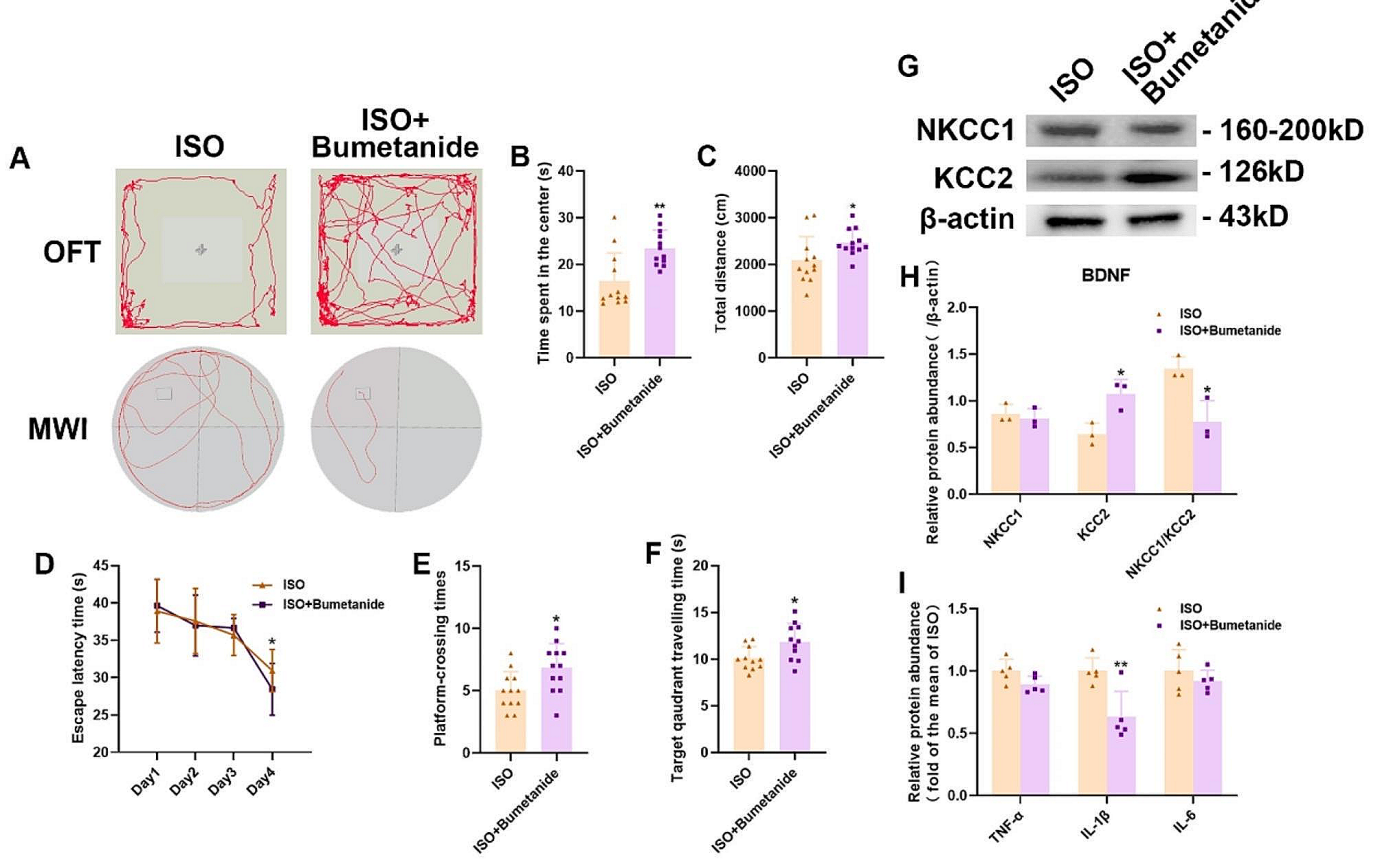
**A** 1. Movement trajectories of mice in the ISO and ISO + Bumetanide groups were analyzed in the OFT and WMZ. **B** The time spent by both groups in the center of the OFT was measured. **C** Total distance covered by mice in the OFT was recorded for both groups. **D** The Escape time on day 4 in the ISO + bumetanide group was significantly shorter compared to ISO group. **E** The platform crossing times in MWM test of the ISO + bumetanide group was significantly longer compared to ISO group. **F** Escape latency time, which indicates how long it took for both groups to find an escape route, was measured during MWM navigation tests, in the ISO + bumetanide group was significantly longer compared to ISO group. **G** Representative western blot bands were used to analyze protein levels of NKCC1 and KCC2 in hippocampus samples. **H** Analysis of NKCC1 and KCC2 protein expression levels was conducted on hippocampus samples. **I** Serum ELISA results showed immune factor IL-1β in ISO was higher than ISO + Bumetanide group on day 34 after birth. All data are presented as mean ± SEM (*n* = 3 ~ 12 per group), with statistical significance denoted as ∗*P* < 0.05 or ∗∗*P* < 0.01 when comparing between ISO and ISO + Bumetanide

Our aforementioned experiments have substantiated alterations in the NKCC1/KCC2 protein ratio in mice subsequent to isoflurane anesthesia, thereby suggesting a pivotal regulatory role of chloride ion transporters in the enduring cognitive decline observed in neonatal rats induced by isoflurane. Consequently, we further investigate the potential efficacy of NKCC1 specific inhibitors (bumetanide) for ameliorating the cognitive impairment and neuropsychiatric disorders instigated by isoflurane. Figure 7.

## Discussion

General anesthetics have the potential to be neurotoxic during brain development, leading to increased apoptotic cells, impaired neurogenesis, and neuroinflammation [8–10]. Certain anesthetics used in early life in rodents and primates may impair cognitive function, with lasting effects into adulthood. All types of general anesthetics can penetrate the brain, and evidence suggests that anesthetics like isoflurane can induce neuronal apoptosis, neuroinflammation, and behavioral deficits in later stages of rat life [11–13]. Sedative and anesthetic drugs are commonly administered for procedural sedation and surgical anesthesia in infants and children. Recent clinical studies have started to establish a link between exposure to anesthesia during infancy in humans and subsequent neuropathology as well as cognitive disturbances later in life [14–16]. Although this topic has been extensively researched previously, the mechanisms underlying the adverse consequences of neonatal anesthesia exposure have only recently been thoroughly investigated [17–20].

The Morris water maze (MWM) is widely utilized for assessing the spatial learning and memory abilities of animals, particularly in relation to neurocognitive disorders [21–23]. In the place navigation test, an increase in escape latency indicates a decline in cognitive ability related to spatial memory [24], while an increase in time spent crossing the platform and staying in the target quadrant suggests improved special information processing skills and overall proficiency [25, 26]. Our findings demonstrate that PND28-31 rats exposed to isoflurane exhibit poor performance on both place navigation and exploration tests. Furthermore, our research reveals impaired spatial memory capabilities among isoflurane-treated pups, as evidenced by increased latency in the MWM task, consistent with previous studies.

Features of depression include having a dark mood, being incapable of experiencing pleasure, having thoughts of suicide and experiencing changeable sleep, appetite, sexual desire, and gastrointestinal motility [27, 28]. For rodents, the measurement of behaviors such as depression and anxiety can be achieved through the SPT, FST, and OFT [29]. Based on our OFT data, rats exposed to isoflurane during the neonatal period can suffer depression-like behaviors in adolescence. In particular, the time of staying in the center area of open field box is shortened and the total distance of movement is reduced [30–32].

In summary, after exposure to isoflurane in the neonatal stage, our results found that rats can suffer mixed neurobehavioral disorders such as cognitive damage and affective disorder, moreover, the damage of isoflurane to the memory ability of newborn rats may be long-term.

Proinflammatory cytokines, including TNF-α, IL-6, and IL-1β, are associated with learning/memory impairment [33–36]. Isoflurane can upregulate the levels of IL-6 in the plasma of patients who receive minimally invasive surgery as well as in neuroglioma cells while increasing TNF-α, IL-1β, IL-6, and caspase-3 expression in adult and aged rodent brains. We also found isoflurane-activated TNF-α, IL-6, and IL-1β in neonatal rats exposed. The findings agree with those of previous studies [37–39], and the hippocampus can be easily affected by neuroinflammation derived from anesthesia and surgery.

The cation-chloride cotransporters Na+–K+–2Cl−–1 (NKCC1) and K+–2Cl−–2 (KCC2) play a critical role in regulating neuronal responses to gamma-aminobutyric acid (GABA). NKCC1 promotes GABA excitation in immature neurons, while the expression of KCC2 inhibits GABA maturation. Imbalance between NKCC1 and KCC2 alters GABA neurotransmission, potentially leading to hyperexcitability in neural circuitry and reduced inhibition following neonatal exposure to anesthesia [3, 40, 41]. Our findings demonstrate that 24 h and 48 h after initial exposure, there is a significant increase in the hippocampal NKCC1/KCC2 ratio due to dysregulated KCC2 expression in isoflurane-exposed pups. This may serve as a crucial factor contributing to cognitive impairment observed in neonatal rats exposed to isoflurane.

Brain-derived neurotrophic factor (BDNF) essentially impacts neuronal plasticity, learning, and memory. Reports have shown that the negative regulatory effect of IL-1β on learning and memory is dependent on neurodegenerative diseases [42–44]. Anesthesia and surgery can trigger microglial activation, IL-1β release, and BDNF downregulation in the hippocampus, thereby leading to hippocampus-dependent cognitive damage in aged mice [45]. The neuroinflammation induced by microglia and astrocytes markedly affects POCD development. In addition, BDNF signaling changes are capable of causing synaptic dysfunction related to memory deficits in Alzheimer disease (AD), Parkinson’s disease (PD), stroke, and sepsis-related encephalopathy [46, 47]. ERK acts as a basic signal for learning and memory [48]. To build long-term memory, ERK signaling is activated in the early stage in the hippocampus [49, 50]. The brain-derived neurotrophic factor precursor-ERK axis in the brain is capable of relieving the anxiety of preadolescent mice exposed to isoflurane after foot-shock stress [51, 52]. Isoflurane inhibited p-ERK expression in both neonatal and adulthood exposures in mice [53, 54].

Our study suggests that exposure to isoflurane in early life can lead to cognitive damage and neuropsychiatric disorders, which may be mediated by decreased levels of BDNF and ERK signaling. During the early stages of development, GABA mediates depolarization excitation through the high expression of NKCC1 ion channels on neurons, maintaining high intracellular chloride ion (Cl-) concentrations. As development progresses, there is an increase in the expression of KCC2 ion channels responsible for pumping Cl- on neurons while the expression of NKCC1 gradually decreases. This shift leads to a transition in GABA-mediated function from excitation to inhibition, which is crucial for synaptic development and neuronal plasticity. Abnormalities in this transformation are associated with various neurological diseases [55, 56].

Our study found that cognitive impairment caused by isoflurane may be related to NKCC1/KCC2. Therefore, we further investigated the potential of using bumetanide as a specific inhibitor for NKCC1 to reverse the cognitive damage and neuropsychiatric disorders induced by isoflurane exposure. We observed that bumetanide effectively reversed the cognitive damage and behavioral disorders induced by isoflurane in neonatal rats through its ability to inhibit TNF-α activation, IL-6 activation, IL-1β activation downregulate NKCC2 expression as well as downregulate BDNF and ERK signaling.

In summary, neonatal exposure to isoflurane during the neurodevelopmental surge elicits behaviors such as anxiety and depression and induces cognitive impairment in adolescent rats, potentially attributed to its detrimental effects on NKCC1 and KCC2. Nonetheless, the mechanism through which NKCC1/KCC2 regulates cognitive impairment may be associated with the expression of BNDF and P-ERK signaling pathways as well as hippocampal inflammatory response. Therefore, it is crucial to identify viable therapeutic strategies for addressing aberrantly activated proinflammatory cytokines or cation-chloride cotransporters that could aid in effective neurotoxic interventions. To extrapolate our findings to a clinical setting, more rigorous epidemiological studies along with clinical trials are urgently required to evaluate the long-term impact of neonatal anesthesia on infants’ development. Discovering insightful mechanisms and developing novel drugs for preventing long-term damage in children is of utmost importance. Our research alone is insufficient; further investigation into specific mechanisms remains superficial.

### Electronic supplementary material

Below is the link to the electronic supplementary material.

Supplementary Material 1

Supplementary Material 2

Supplementary Material 3

## Data Availability

All the data supporting our findings is contained within the manuscript.
