# Supplementary material for: Isoflurane-induced neuroinflammation and NKCC1/KCC2 dysregulation result in long-term cognitive disorder in neonatal mice
Source: BMC Anesthesiol. 2024 Jun 5;24:200. doi: 10.1186/s12871-024-02587-6 (PMC11151488; doi:10.1186/s12871-024-02587-6)
Supplement: Supplementary file 1 — Supplementary Material 1 [file 12871_2024_2587_MOESM1_ESM.pdf]

中山大学动物实验伦理审查同意书

Affidavit of Approval of Animal Use Protocol, IACUC, SYSU

申请编号 Application No.	2021001818	批准编号 Approval No.	SYSU-IACUC-2022-000097

本动物实验方案经过中山大学实验动物伦理委员会审核，符合动物保护、动物福利和伦理原则，符合国家实验动物福利伦理的相关规定。The animal use protocol listed below has been reviewed and approved by the Institutional Animal Care and Use Committee (IACUC), Sun Yat-Sen University.

实验名称 Protocol Title	异氟醚通过氯转运体 NKCC1/KCC2途径致新生 SD 小鼠神经炎症和长期认知功能障碍<br>Isoflurane administered to neonatal SD rats causes neuroinflammation and long-lasting cognitive impairment through Chloride Transporter NKCC1/KCC2 way				
实验申请人 Applicant	洪玉<br>hongyu	职称/学位 Title/Degree	主治医师<br>Attending Doctor	邮箱 Email	184494780@qq.com
实验负责人 Principle Investigator (PI)	曹林<br>CAOLIN	职称/学位 Title/Degree	主任医师<br>Chief Physician	邮箱 Email	caolin@mail.sysu.edu.cn
院系(部门) Department	中山大学孙逸仙纪念医院（附属第二医院）<br>SUN YAT-SEN MEMORIAL, SUN YAT-SEN UNIVERSITY	申请日期 Application date		2021/12/3	
动物种系 Species or Strains	大鼠 CD(SD)IGS<br>CD(SD)IGS	动物数量 Quantity		70	
计划执行时间 Period of Protocol	2020/6/28 ~2021/12/3	实验动物使用许可证 Number of Animal use permit		中山大学（实验动物中心北校园）（SYXK（粤）2017-0081）	
审查意见 Results of inspection	☑符合动物福利伦理要求，同意实验 **Agree**<br>☐调整方案后，可进行实验 **Agree after modification**				
兽医师 Chief Veterinary Officer	郭中敏 (signature)	日期 Date		2022-01-13	

中山大学实验动物管理与使用委员会 IACUC SYSU

中山大学实验动物伦理委员会（IACUC，SYSU）

主席（Chairman）： (signature)

日期（Date）： 2022-01-13
