# Supplementary figures and images for: Isoflurane-induced neuroinflammation and NKCC1/KCC2 dysregulation result in long-term cognitive disorder in neonatal mice
Source: BMC Anesthesiol. 2024 Jun 5;24:200. doi: 10.1186/s12871-024-02587-6 (PMC11151488; doi:10.1186/s12871-024-02587-6)

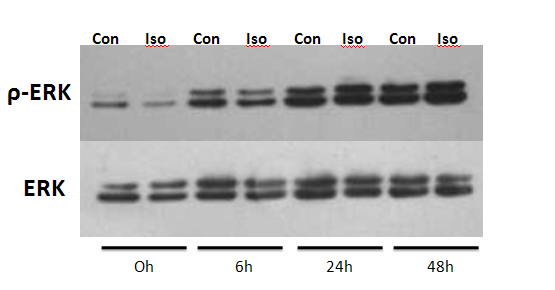

Supplement: Supplementary file 3 — Supplementary Material 3 [file 12871_2024_2587_MOESM3_ESM.docx]
